# circPVT1 regulates EMT and induces macrophage polarization to promotes the progression of renal cell carcinoma

**DOI:** 10.3389/fimmu.2026.1760058

**Published:** 2026-04-21

**Authors:** Minhao Zhang, Aifeng He, Zilong Xu, Ming Chen, Houliang Zhang, Weipu Mao

**Affiliations:** 1 Department of Urology, Zhongda Hospital Southeast University, Xishan People’s Hospital of Wuxi City, Wuxi, China; 2 Emergency Department, Binhai County People’s Hospital, Yancheng, China; 3 Department of Radiation Oncology, The First Affiliated Hospital of USTC, Division of Life Sciences and Medicine, University of Science and Technology of China, Hefei, China; 4 Department of Urology, Nanjing Lishui District People’s Hospital, Zhongda Hospital Lishui Branch, Southeast University, Nanjing, China; 5 Department of Urology, Affiliated Zhongda Hospital of Southeast University, Nanjing, China

**Keywords:** circPVT1, EMT, macrophage polarization, renal cell carcinoma, single-cell sequencing

## Abstract

**Introduction:**

Renal cell carcinomas (RCC) are resistant to chemotherapy and radiotherapy, and effective treatment options remain limited. Immunotherapy has emerged as a promising approach, and circular RNAs (circRNAs) are increasingly recognized as key regulators of tumor immunity. This study aims to elucidate the regulatory relationship between circPVT1 and antitumor immunosuppression in RCC.

**Methods:**

CircPVT1 expression and its prognostic value were analyzed in RCC samples. The effects of circPVT1 knockdown on RCC cell proliferation, invasion, and metastasis were examined *in vitro*. Singlecell sequencing was used to assess macrophage infiltration in circPVT1high versus circPVT1low groups. ELISA was performed to measure secretion of IL4, IL10, and TGFβ by tumor cells. In addition, nanotherapeutic system delivering circPVT1 inhibitors was tested for its antitumor efficacy.

**Results:**

CircPVT1 was highly expressed in RCC and correlated with poor prognosis. Knockdown of circPVT1 significantly suppressed cell proliferation, invasion, and metastasis. Singlecell sequencing revealed increased macrophage infiltration in the circPVT1high group. ELISA results demonstrated that circPVT1 knockdown reduced tumor cell secretion of IL4, IL10, and TGFβ, thereby promoting macrophage polarization toward the M1 phenotype. Mechanistically, circPVT1 promoted RCC progression by regulating EMT. Furthermore, a nanotherapeutic system containing circPVT1 inhibitors effectively inhibited RCC growth.

**Discussion:**

These findings indicate that circPVT1 plays a critical role in RCC immune evasion by modulating macrophage polarization and EMT. Therefore, circPVT1 may be a predictor of ccRCC immune evasion and a potential therapeutic target.

## Introduction

Renal cell carcinoma (RCC) as the common cancers in adults contributed approximately 3% of systemic malignancies tumors (1). Clear cell renal cell carcinoma (ccRCC) was the most common subtype and accounted for 80%-90% of RCC (2). For localized RCC patients, partial nephrectomy was the first choice for treatment (3). However, 30% of patients will recur after initial surgical resection (4). For patients with recurrence and metastasis, radiotherapy and chemotherapy were less effective. Tyrosine kinase inhibitors (TKIs) represent a cornerstone in the management of advanced or metastatic RCC, yet acquired resistance ultimately limits their efficacy (5). Emerging evidence demonstrates that immune checkpoint inhibitors (ICIs) have achieved significant success in treating patients with ccRCC, highlighting the immense potential of immunotherapy (6). Nevertheless, persistent challenges such as low response rates and adaptive immune resistance necessitate further investigation into the tumor immune microenvironment to identify novel therapeutic targets. Therefore, it is urgent to explore the molecular mechanisms underlying the development of ccRCC and therapeutic targets which will benefit patients.

Single-cell RNA sequencing (scRNA-seq) is a powerful technology that enhances bulk RNA-seq by providing transcriptional insights at the single-cell level (7). scRNA-seq enables high-resolution deconvolution of tissue-specific gene expression signatures at the cellular level, thereby unraveling cellular heterogeneity and differentiation (8). However, scRNA-seq requires organs and tissues to be dissociated into single-cell suspensions, which inevitably leads to the loss of spatial localisation information (9, 10). In contrast, spatial transcriptomics (ST) is a widely used tool for studying the cancer microenvironment that preserves the spatial distribution of gene expression profiles. ST enables the visualization and quantitative analysis of tissue sections, a capability not available in traditional scRNA-seq methods (11). ScRNA-seq and ST have complementary advantages: they not only systematically identify cell subtypes within tissues but also reveal spatial position, significantly expanding research methods across multiple disease domains (12). Recently, Song et al. utilized spatial and single-cell transcriptomics to unveil heterogeneity in ccRCC, confirmed that a distinct subset of regulatory T cells (Tregs) expressing multiple cytokines established spatial colocalization with tumor-associated macrophages (TAMs) at the tumor-normal interface, forming a positive feedback loop and maintaining a synergistic procarcinogenic effect (13). To elucidate the tissue structure and molecular landscape of ccRCC, this method combines scRNA-seq with ST to explore the multi-layered and multi-dimensional characteristics of intercellular communication and potential progression mechanisms.

Circular RNAs (circRNAs) represent a class of evolutionarily conserved, ubiquitously expressed noncoding RNAs characterized by their covalently closed-loop structure, which confers exonuclease resistance (14). CircRNAs are involved in the regulation of transcription and splicing processes, the influence of mRNA stability and translation, and the regulation of protein function and metabolism (15). They can also translate functional peptides via IRES/m6A mechanisms in a variety of biological and pathophysiological environments (16). In addition, circRNA participates in the regulation of tumor cells and the tumor microenvironment (TME), thereby influencing anti-tumor immunity (17). However, only a few studies have reported the expression profiles of circRNAs associated with immunosuppressive signaling, specifically with regard to macrophage polarization in ccRCC.

In our previous work, we demonstrated that circpvt1 functions as a circular RNA in renal cell carcinoma (18). In this study, we demonstrated that circPVT1 (hsa_circ_0001821) is upregulated in ccRCC, and its knockdown inhibits tumor progression and enhancing immunosuppression. Mechanistically, circPVT1 knockdown upregulates E-cadherin, and downregulated N-cadherin and vimentin. Knockdown of circPVT1 induces macrophage polarization toward the M1 phenotype. Moreover, we constructed the liposome-encoding si-circPVT1, and prepared a novel si-circPVT1@Liposomal Polydopamine@MUC12 nanoparticles (SCLPM-NPs) to targeted deliver si-circPVT1 to the ccRCC cell lines by the incorporation of MUC12 anti-body into polydopamine (PDA). The results indicated that SCLPM-NPs can effectively target tumor tissue sites and inhibit tumor growth.

## Materials and methods

### Clinical specimens

ccRCC tissues were obtained from 6 patients who underwent nephrectomy at Zhongda Hospital, Southeast University (Nanjing, China) between January 2024 and June 2025. Following surgical resection, the tissues were frozen immediately and stored at -80 °C. The study protocol was reviewed and approved by the Ethics Committees of Zhongda Hospital, Southeast University. All patients provided written informed consent.

### Cell culture and lentivirus transfection

HK-2, 786-O, ACHN, OSRC-2 and A498 purchased from the Cell Bank of the Chinese Academy of Sciences (Shanghai, China). Keratinocyte Serum Free Medium (K-SFM, Gibco, USA) was used to culture HK-2 cells and OSRC-2 cell lines were cultured in RMPI-1640 medium (Gibco, USA). A498 cell lines were cultured in MEM (Gibco, USA) and other cells were cultured in Dulbecco’s Modified Eagle’s Medium (DMEM, Gibco, USA). All cell lines were supplemented with 10% foetal bovine serum (FBS) (Epizyme, China) and 1% penicillin/streptomycin (P/S) (NCM Biotech, China). All cell lines were cultured at 37 °C in a conventional incubator containing 5% CO_2_.

### Macrophage culture and treatment

Macrophages were differentiated from THP-1 cells, a human monocytic leukemia cell line kindly provided by Guangzhou ELGBIO Company. THP-1 cells were cultured in RPMI-1640 medium supplemented with 10% FBS at 37 °C in a humidified 5% CO_2_. To differentiate THP-1 cells into macrophages, THP-1 cells were cultured with phorbol 12-myristate 13-acetate (PMA; 100 ng/mL; MCE) in RPMI-1640 medium without FBS for 48 h.

### Co-culture experiments

We collected the media from different groups and centrifuged the conditioned medium at 12,000 rpm to remove debris. Before treating THP-1 cells, the supernatant was collected and diluted twice with a fresh medium. THP-1 cells were then harvested for further analysis after 48 h of incubation.

### Western blot and Quantitative real-time PCR

First, the cells were lysed on ice with RIPA lysis buffer (YEASEN, China) for half an hour and sonicated once. The supernatant was collected by centrifugation for protein quantification. Proteins (30 ug) were added into SDS-polyacrylamide gel and the target proteins were transferred to PVDF membranes. The membranes were incubated with primary antibodies and secondary antibodies (Abcam, USA) and were visualized by ECL. Total RNA was extracted from cells or tissue specimens using the Trizol reagent (TaKaRa). The concentration of all samples was determined via NanoDrop 2000, and the relative expression was calculated using the 2^-ΔΔCt^ method.

### Colony formation, EdU assay, wound healing and transwell assay

For colony formation assay, 5×10^2^ cells were cultured in 6-well plates then stained and observed after 2 weeks. Cells were cultured for 2h with 10 μM EdU (Beyotime, China) and stained following the operating instructions. EdU results were photographed using Leica microscope system (Leica, Germany). The sterile 200 μL tips were used to make the wound and recorded at 0h and 24h. For the transwell assay, 5×10^4^ cells were cultured in upper chambers without FBS and collected after 24 hours.

### Immunohistochemistry staining

Briefly speaking, fresh tumor tissues fixed in 4% paraformaldehyde. After ethanol dehydration, the tissue was embedded in paraffin. The tissue was sliced into 5 μm slices and incubated with primary antibodies at 4 °C overnight after blocking with bovine serum albumin (BSA). Then, the tissues were photographed using the microscope (Leica Microsystems, Germany) after incubated with secondary antibodies.

### Xenograft tumor model

All animals were randomly assigned to the control and experimental groups. Each mouse was seeded 1×10^6^ tumor cells after adaptive feeding. The size of the tumor was measured every week. D-luciferin potassium salt solution (100 μg/g, Beyotime) was injected into the abdomen of all mice to emit luminescence. After 15 minutes, the luminescence intensity was measured using an *in vivo* imaging system (AniView100, BLT, Guangzhou, China).

### Synthesis of si-circPVT1@Liposomal PDA@MUC12 nanoparticles

The si-circPVT1@Liposomal PDA@MUC12 nanoparticles (SCLPM-NPs) were synthesized by a rapid and green method. Briefly, 50 nM si-circPVT1 dissolved in RNase free water was loaded into the commercial liposomes (50 μL; Yeasen, China) by vortexing for 30 s to form si-circPVT1@lipsome. The resulting suspension was dispersed in Tris–HCl (pH 8.8) solution, followed by the addition of dopamine hydrochloride (5 mg). After stirring for 3 hours, PDA-modified liposomes were obtained. Then, the PDA-modified liposomes were dispersed in streptavidin solution (2 mg/mL) and shaken thoroughly in dark at 4 °C resulting in the synthesis of si-circPVT1@Liposomal PDA @Str. For SCLPM-NPs synthesis, the obtained mixtures were mixed with biotinylated MUC12 antibody solution (Bioss, China) and incubated for 1 h. The obtained suspension was washed using PBS buffer and stored at 4 °C till further use.

### Single-cell RNA-sequencing

Referring to our previously published article (19), single-cell RNA sequencing analysis was performed following the steps of single-cell suspension preparation, single-cell RNA sequencing, data preprocessing and quality control, batch effect correction and dimensionality reduction, and cell type annotation. Macrophages were marked with CSF1R, C1QA, and C1QB; neutrophils with S100A8, S100A9, and CSF3R; T cells with CD3D, CD3E, and CD3G; epithelial cells with KRT18, KRT19, and EPCAM; endothelial cells with VWF, PECAM1, and CLDN5; B cells with CD19, CD79A, and MS4A1; fibroblasts with COL1A1, COL1A2, and PDGFRA; and plasma cells with JCHAIN and TNFRSF17.

### Enzyme linked immunosorbent assay

The levels of human IL-4, IL-10, and TGF-β in the culture medium were measured according to the instructions of the ELISA kit (Ruixinbio, Quanzhou, China) for the corresponding indicators. The optical density (OD) of the samples was measured at 450 nm using a microplate reader (Epoch, BioTek), and the concentrations of each sample were calculated based on the standard curve.

### Statistical analysis

Statistical analyses were performed using SPSS 23.0 software (IBM, USA) and GraphPad Prism 10 software. The experimental data were analyzed using Image J. Data were presented as mean ± standard deviation (SD). The two-tailed student t-test was used to compare parameters between two groups of normally distributed data. For comparisons among multiple groups, one-way or two-way analysis of variance (ANOVA) was used, followed by Tukey’s *post hoc* test for pairwise comparisons when significant differences were detected. Statistical significance was defined as P < 0.05.

## Result

### CircPVT1 promoted proliferation, migration, and invasion of ccRCC cells

**Figure 1:**
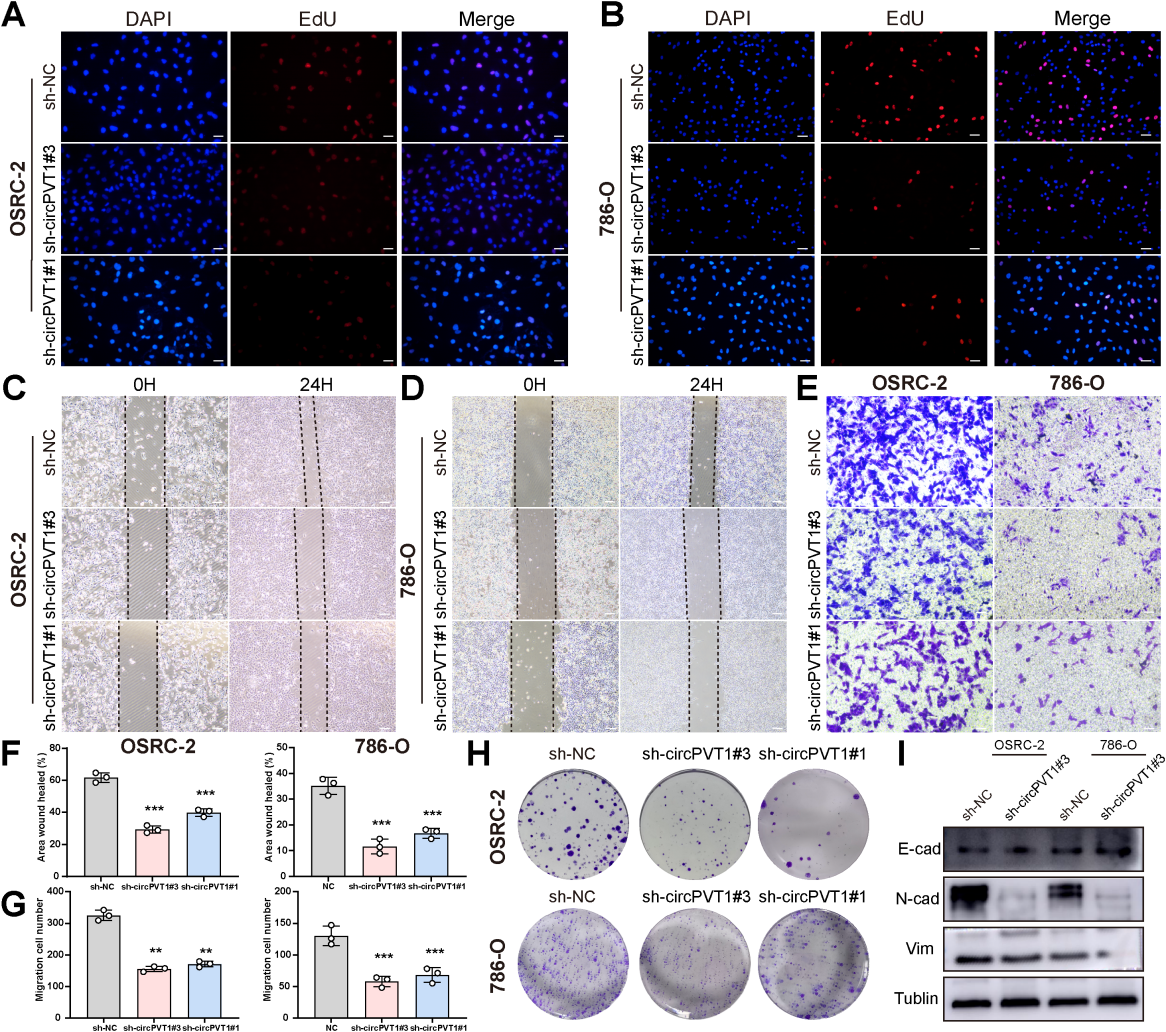
**(A, B)** The proliferation of OSRC-2 and 786-O cells treated with sh-circPVT1#3, sh-circPVT1#1 or sh-NC was assessed by the EdU assay. **(C, D)** Wound healing assays were performed in OSRC-2 and 786-O cells treated with sh-circPVT1#3, sh-circPVT1#1 or sh-NC. **(E)** Transwell assays were performed in transfected OSRC-2 and 786-O cells to assess cell migration capacity. **(F, G)** The corresponding quantitative analysis of wound healing assays **(F)** and transwell migration assays **(G)** in OSRC-2 and 786-O cells. **(H)** Effect of circPVT1 on proliferation in OSRC-2 and 786-O cell lines was determined through colony formation assay. **(I)** Western blotting of vimentin, N-cadherin, and E-cadherin in OSRC-2 and 786-O cells treated with circPVT1 knockdown or negative control. Scale bar: 100 μm. Statistical significance is indicated (**P<0.01, ***P<0.001) by Student’s t-test or ANOVA.

First, we analyzed the circPVT1 expression in ccRCC cell lines. The expression of circPVT1 in tumor cell lines (786-O, OSRC-2, ACHN and A498) was significantly higher than that in HK-2 cells (**Supplementary Figure 1**). Our next step was to examine how circPVT1 played a role in ccRCC progression. Then, Real-time PCR was employed to validate the transfection efficiency (**Supplementary Figure 2**). Thus, we chose to establish circPVT1 stable knockdown (choose the high efficiency sh-circPVT1#1 and sh-circPVT1#3) in 786-O and OSRC-2 (which has more endogenous circPVT1). Functionally, the proliferation potential of sh-circPVT1 cells in 786-O cells and OSRC-2 cells was significantly reduced compared with controls according to EdU assay (**Figures 1A, B**; **Supplementary Figure 3**). Likewise, similar results had been observed in wound-healing experiments and the migration capability of sh-circPVT1-transfected cells was reduced in 786-O cells and OSRC-2 cells (**Figures 1C, D, F**). Moreover, circPVT1 knockdown reduced migration and invasion ability of 786-O cells and OSRC-2 cells comparing the control group (**Figures 1E, G**; **Supplementary Figure 4**). According to colony formation assay results, lower levels of circPVT1 markedly decreased colony formation (**Figure 1H**; **Supplementary Figure 5**). Previous studies have indicated that most ccRCC originate from renal tubular epithelial cells, and epithelial-mesenchymal transition (EMT) is a key event in the invasion and metastasis of epithelial-derived cancers (20). Therefore, we subsequently used WB to verify the expression of EMT markers. The results indicated that E-cadherin, which epithelial marker, were increased, whereas the levels of mesenchymal markers including N-cadherin and Vimentin were decreased in 786-O and OSRC-2 sh-circPVT1 cells (**Figure 1I**).

### Knockdown circPVT1 inhibits the growth and metastasis of ccRCC *in vivo*

**Figure 2:**
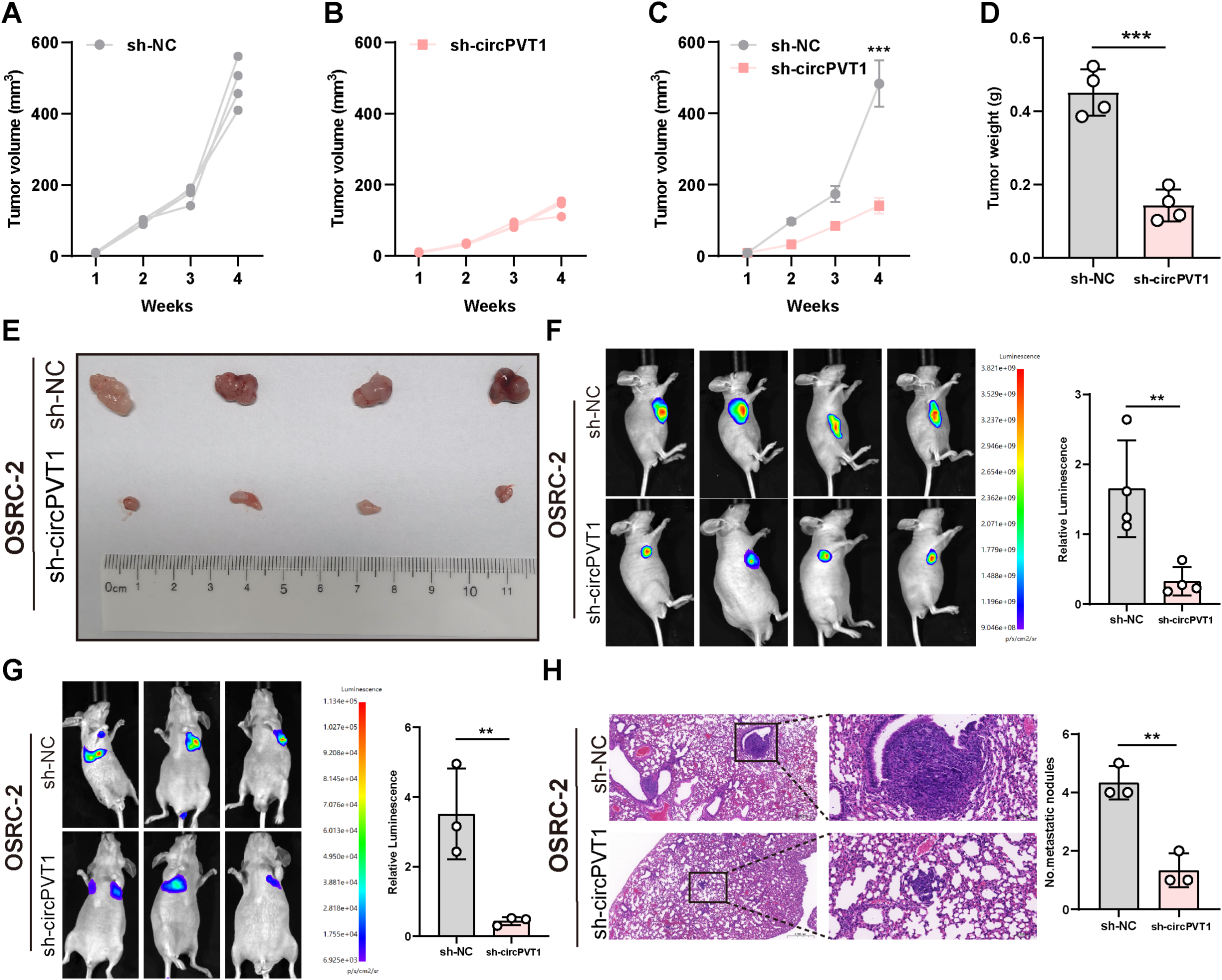
**(A–C)** Growth curves of xenografts in control and sh-circPVT1 groups. **(D)** Average tumor weight collected from different groups. **(E)** Representative images of the excised tumors after different treatments. **(F)** IVIS imaging of subcutaneous xenograft tumors. **(G)** Representative images of metastasis observed through IVIS. **(H)** H&E images of lung tissue sections from the control and sh-circPVT1 groups. Scale bar: 100 μm. **P<0.01, ***P<0.001.

The above *in vitro* experiments preliminarily validated the tumor promoter effect of circPVT1. Subsequently, we constructed OSRC-2 cells with stable circPVT1 knockdown and explored the function of circPVT1 *in vivo*. Nude mice were randomly assigned to control and experimental groups. Then, the transfected cells were subcutaneously implanted into nude mice. After four weeks of treatment with knockdown of circPVT1, the tumor volume was lower than that of the control group (**Figures 2A–C**). Tumor weights and the respective digital images revealed the sh-circPVT1 group had a more significant tumor-suppressing effect compared with the control groups (**Figures 2D, E**). As demonstrated by *In Vivo* Imaging Systems (IVIS), circPVT1 knockdown dramatically reduced tumor proliferation (**Figure 2F**). To further explore the effects of circPVT1 in tumor metastasis, OSRC-2 cells with stable circPVT1 knockdown were injected into tail vein of nude mice. As expected, decreased luciferase signal was found in sh-circPVT1 group, showing circPVT1 knocked-down inhibited ccRCC cell metastases (**Figure 2G**). HE analysis also confirmed the number of pulmonary nodules metastasis was reduced compared to the control group (**Figure 2H**).

### CircPVT1 promotes M2 subtype polarization

**Figure 3:**
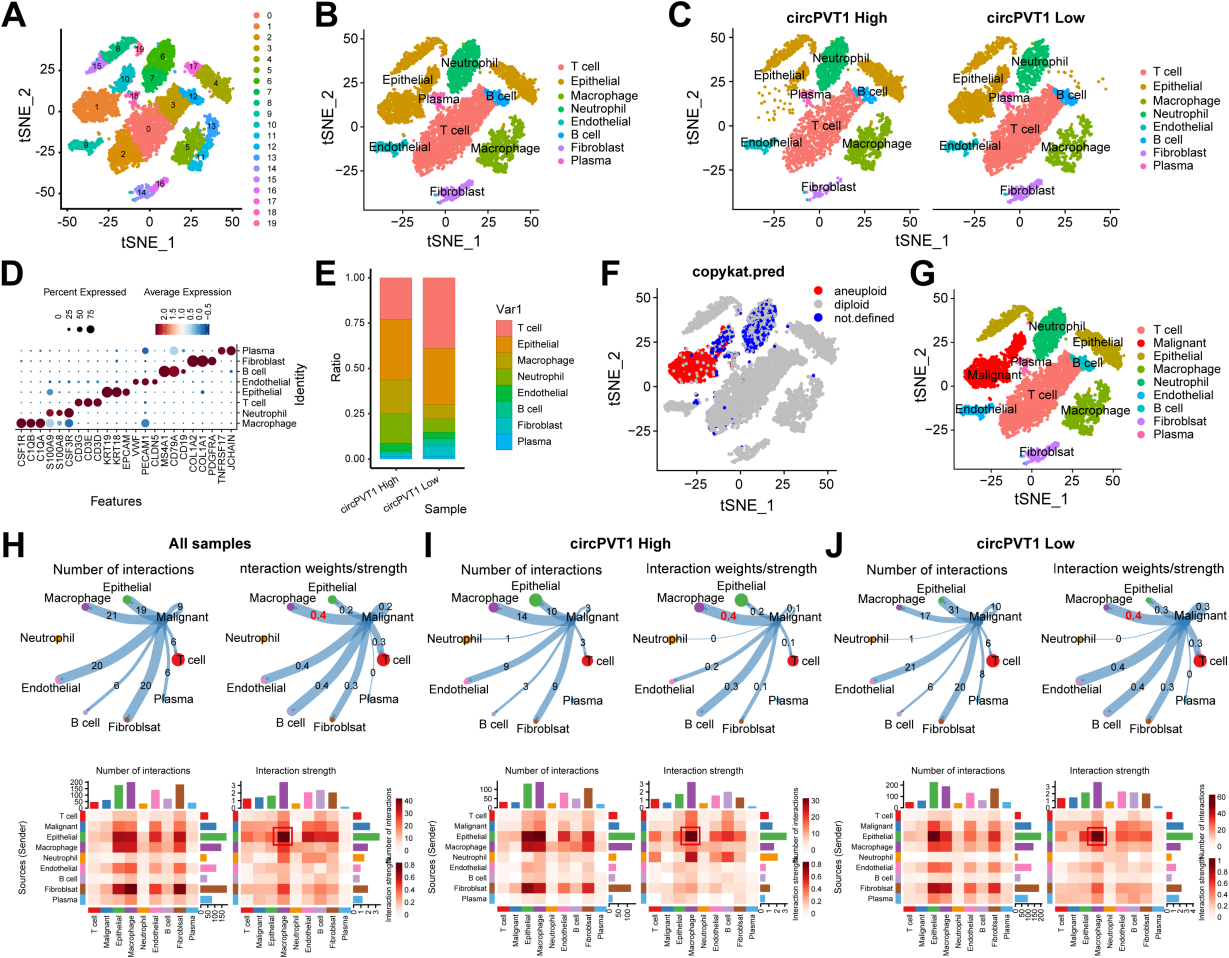
**(A)** t-SNE plots of the 20 clusters. **(B)** t-SNE plots of the 8 cell types. **(C)** Distribution maps of 8 major cell types in circPVT1 high and circPVT1 low patients. **(D)** Bubble diagram of canonical marker gene expression across 8 major cell types. **(E)** Percentage of cells in samples for 8 cell types. **(F)** copykat distinguishes malignant tumor cells and normal epithelial cells. **(G)** t-SNE plots of the 9 cell types. **(H)** Cell-cell communication results for malignant tumor cells and other cell types in all samples. **(I)** Cell-cell communication results for tumor cells and other cell types in circPVT1 high sample. **(J)** Cell-cell communication results for tumor cells and other cell types in circPVT1 low sample.

To investigate the *in vivo* effects of circPVT1, we collected tumor samples from six RCC patients with different circPVT1 expression levels for single-cell sequencing. Prior to conducting single-cell analysis, we analyzed the expression of circPVT1 in six samples using qPCR. The grouping was determined by setting a threshold at the median expression value. Based on these expression levels, samples were categorized into two groups, the circPVT1 high group (samples 1, 4, and 6; n = 3) and the circPVT1 low group (samples 2, 3, and 5; n = 3) (**Supplementary Figure 6**). After integrating the transcriptional data from all cells, low-resolution t-distributed stochastic neighbor embedding (t-SNE) clustering analysis identified 20 clusters within the samples (**Figure 3A**). Using well-known markers, we classified the 20 cell clusters into 8 major cell types from six RCC patients (**Figures 3B–D**). Comparative analysis between patients with high and low circPVT1 expression revealed an increase in macrophages, neutrophils, and endothelial cells in patients with high circPVT1 expression, whereas T cells, B cells, and fibroblasts cells were decreased (**Figure 3E**). Subsequently, we showed the distribution of maligant epithelial cells and normal epithelial cells by copykat analysis (**Figures 3F, G**). We examined the cell-cell communication relationships between malignant cells and other cell types and found that the number of interactions between malignant cells and macrophages, fibroblasts, and endothelial cells was higher in all samples (**Figure 3H**). However, the interaction strength was the strongest between malignant cells and macrophages (**Figure 3H**). We also analyzed the communication relationships between malignant cells and other cells within the samples. In the circPVT1 high sample, the interaction strength between malignant cells and macrophages was significantly increased compared with circPVT1 low sample (**Figures 3I, J**).

**Figure 4:**
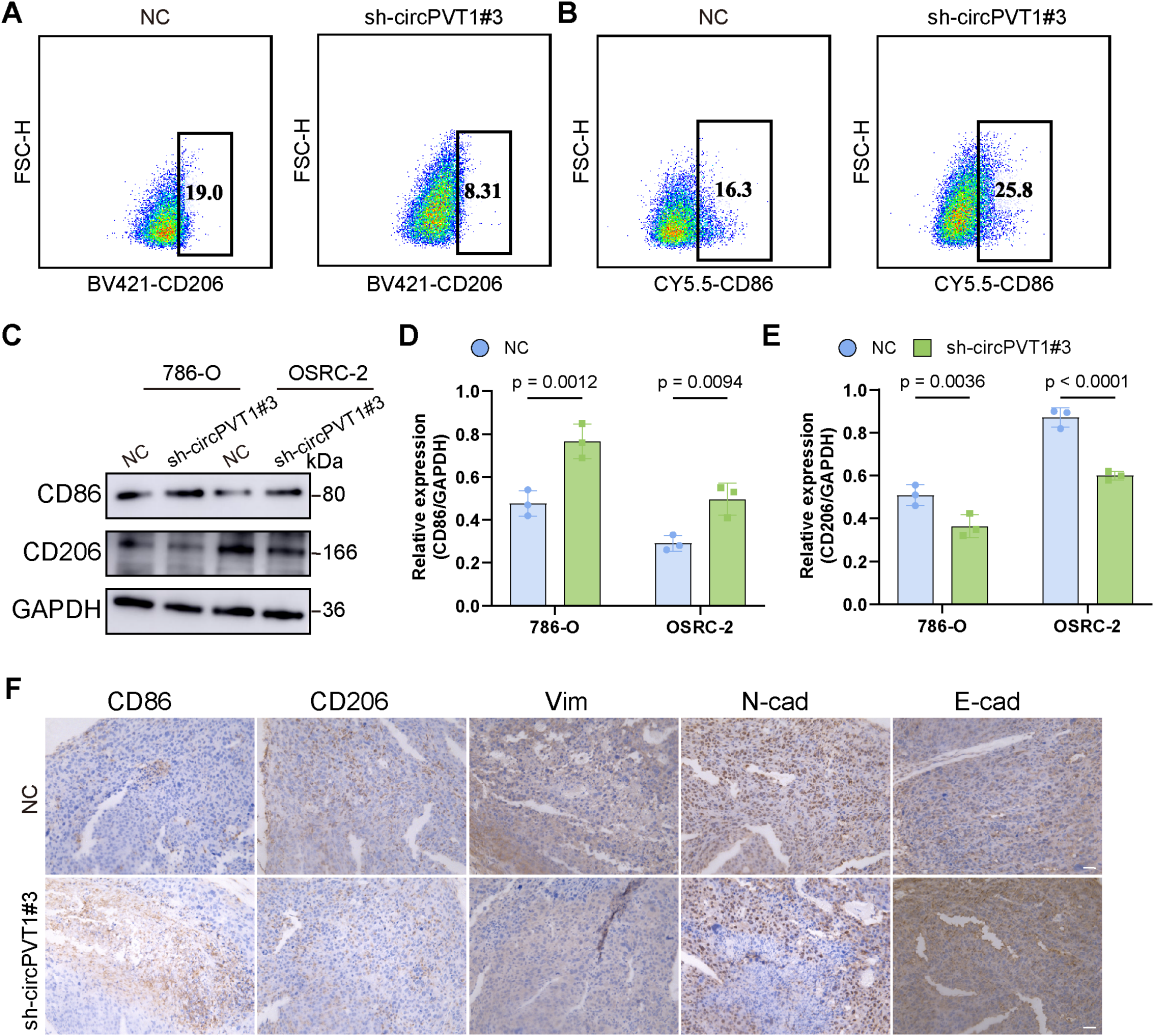
**(A, B)** Representative flow cytometry plots show the percentage of M2 and M1 macrophage. **(C–E)** Western blotting was performed to verify the expression of CD86 and CD206 in the NC and sh-circPVT1 groups. **(F)** IHC staining of CD86, CD206, Vim, N-cad, and E-cad in the NC and sh-circPVT1 groups. Scale bar: 100 μm.

Tumor-associated macrophages (TAMs) play a key role in maintaining tumor growth, invasiveness, metastasis, and responsiveness to drugs (21). M2 macrophages promote tumor growth, angiogenesis, and metastasis, and they are associated with malignant progression (22). Compared with patients with renal cancers infiltrated with low levels of M2 macrophages, those with high M2 macrophage infiltration have poorer overall and progression-free survival (23). This suggests that M2 macrophages serve as potential biomarkers and promising immunotherapeutic targets for renal cancer. Thus, we assessed whether circPVT1 could influence the polarization of macrophages. After incubation with the conditioned medium, the macrophages were collected for flow cytometry and WB assays. Compared to the control group, flow cytometry results revealed the M2 phenotype significant reduced, while the M1 phenotype was increased in sh-circPVT1#3 group (**Figures 4A, B**). WB results indicated that CD206 expression was decreased in the sh-circPVT1#3 group, whereas CD86 expression was elevated (**Figures 4C–E**). Then, we conducted the direct co-culture experiments with circPVT1-knockdown tumor cells, followed by flow cytometry, and the result provide direct evidence that circPVT1 in tumor cells regulates macrophage polarization through paracrine signaling mechanisms (**Supplementary Figure 7**). Studies have shown that macrophage polarization is regulated by cytokines (24). ELISA experiments detected the expression levels of cytokines in the tumor cell culture medium, showing that circPVT1 knockdown significantly reduced the levels of IL-4, IL-10, and TGF-β (**Supplementary Figure 8**). Additionally, the results of IHC staining demonstrated higher expression of N-cadherin, Vimentin and CD206 in the control groups, while CD86 and E-cadherin were highly expressed in the sh-circPVT1#3 group (**Figure 4F**). Collectively, these findings underscore that circPVT1 promotes the conversion of macrophages to the M2 subtype and facilitates ccRCC tumor progression.

### Syntheses and characteristics of SCLPM-NPs

**Figure 5:**
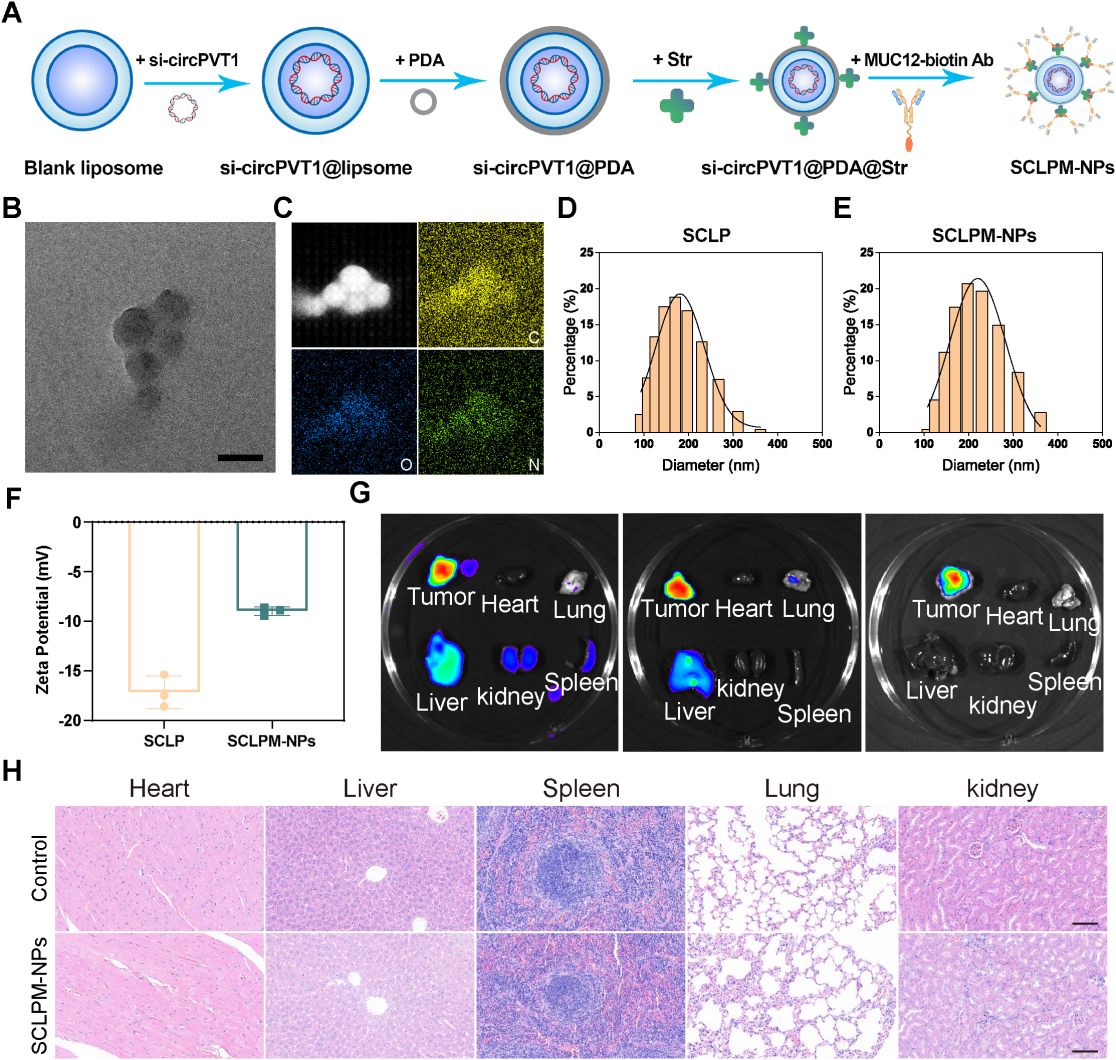
**(A)** Schematic diagram of the SCLPM-NPs Synthesis Process. **(B)** TEM image of SCLPM-NPs. **(C)** Elemental mappings show the distribution of O, C and (N) **(D, E)** Hydrodynamic diameter of **(D)** SCLP and **(E)** SCLPM-NPs. **(F)** Zeta potentials of SCLP and SCLPM-NPs. **(G)** *Ex vivo* fluorescence imaging of the tumor and the major organs including heart, liver, spleen, lung, and kidney. **(H)** HE staining of organs in the control group and SCLPM-NPs group to assess *in vivo* safety. Scale bar: 100 μm.

Liposomal and polydopamine nanoparticle (PDA-NP) drug delivery systems (NDDSs) have emerged as pivotal platforms in precision oncology (16). Therefore, we synthesized SCLPM-NPs for therapy of ccRCC, and the strategy for preparing the SCLPM-NPs is shown in **Figure 5A**. TEM imaging revealed the SCLPM-NPs had a near-spherical shape, and EDS mapping showed the C, N, and O elements, proving the successful synthesis of PDA (**Figures 5B, C**). The decreased zeta potential and increased diameter indicated that MUC12 had been incorporated into the SCLP surface, resulting in stable SCLPM-NPs with an average diameter of approximately 200 nm (**Figures 5D–F**). To evaluate the inhibitory efficiency of our nanoparticle system, we transfected OSRC-2 cells with SCLPM-NPs or, with shcircPVT1. The knockdown efficiency of circPVT1 was assessed by qPCR after 48 hours post-transfection. Our results demonstrate that SCLPM-NPs effectively knocked down circPVT1 expression *in vitro*, achieving a significant reduction compared to the control group (**Supplementary Figure 9**). In order to verify the *in vivo* enrichment effect of SCLPM-NPs, the cy5.5 labeled SCLPM-NPs were injected into nude mice via the tail vein. Fluorescence results demonstrated that the SCLPM-NPs successfully accumulated in the tumor (**Figure 5G**). Before performing animal therapeutic experiments, we initially assessed the safety of SCLPM-NPs *in vivo*. HE results showed that no significant morphological changes were observed in the organs between the SCLPM-NPs group and the control group (**Figure 5H**).

### *In vivo* therapeutic effects of SCLPM-NPs using a subcutaneous tumor model

**Figure 6:**
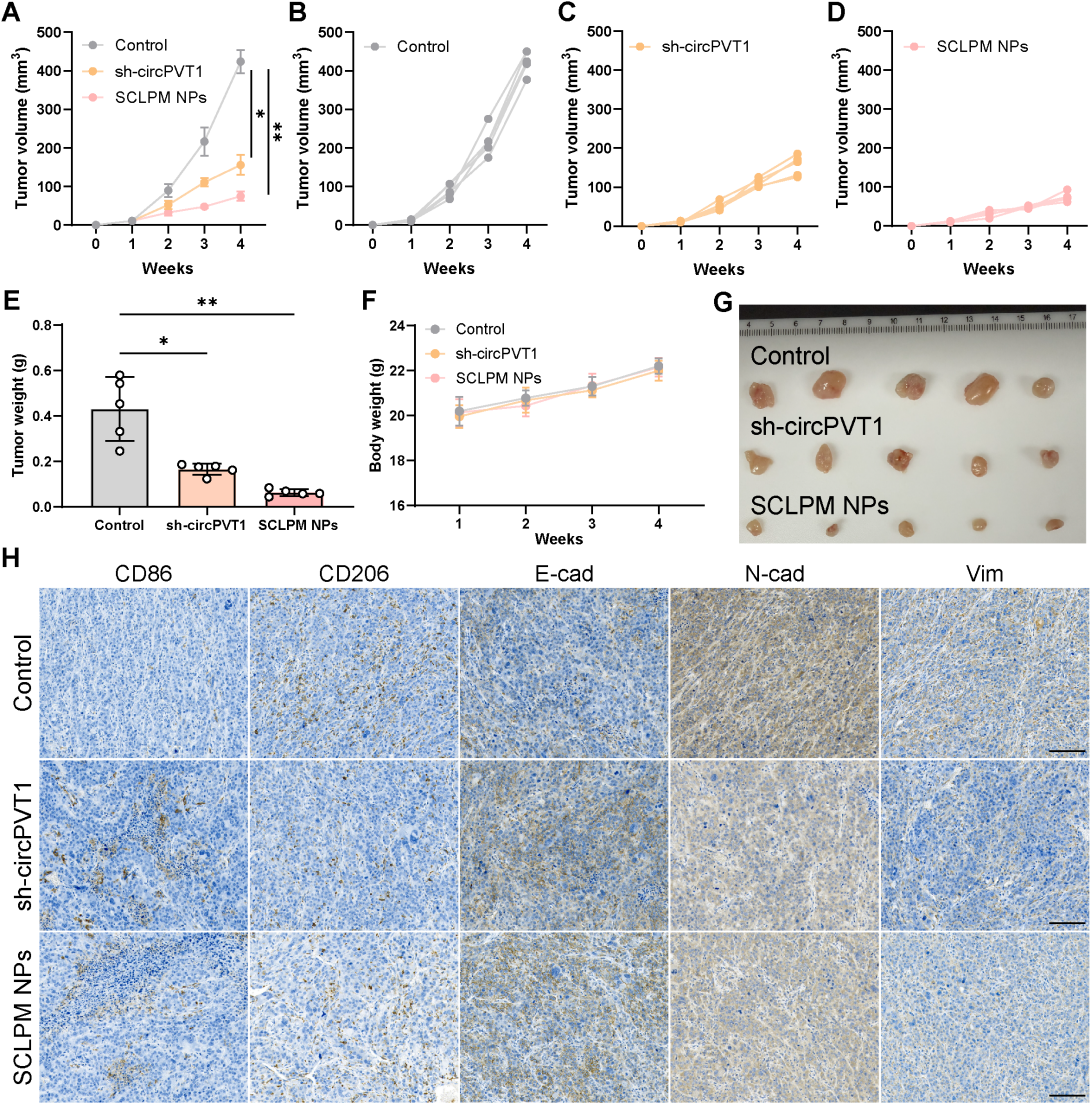
**(A–D)** Growth curves of xenografts. **(E)** Average tumor weight collected from different groups. **(F)** Body weight changes of mice during the treatments (n=5). **(G)** Representative images of the excised tumors after different treatments. **(H)** IHC staining of CD86, CD206, Vim, N-cad, and E-cad in the control, sh-circPVT1 and SCLM NPs groups. Scale bar: 100 μm.

Given the *in vitro and in vivo* experimental results of circPVT1 and the good tumor enrichment effect of SCLPM-NPs, we further explored the *in vivo* treatment of SCLPM-NPs. During treatment, tumor volumes was monitored to evaluate the antitumor efficacy. The SCLPM-NPs groups demonstrated better anti-tumor effects than that of the sh-circPVT1 groups (**Figures 6A–D**). The tumor weight also confirms this (**Figure 6E**). During the treatment period, the mice showed no significant weight loss, further confirming the safety of SCLPM-NPs (**Figure 6F**). Analysis of the digital images revealed that the SCLPM-NPs groups had better tumor inhibition compared to the other groups (**Figure 6G**). Subsequently, IHC staining demonstrated CD86 and E-cadherin were highly expressed in the sh-circPVT1 and SCLPM-NPs groups, while higher expression of N-cadherin, Vimentin and CD206 were found in the control group (**Figure 6H**). In conclusion, SCLPM-NPs suppressed the growth of subcutaneous tumors via macrophage polarization and EMT pathway.

## Discussion

CircRNAs exhibit tissue specific expression and nuclease resistance due to their covalently closed-loop structure (25), enabling their involvement in diverse pathological processes, including cancer (26). Meanwhile, an increasing number of studies have found that circPVT1 plays a significant role in the initiation and progression of various tumors. For example, circPVT1 sponges miR-33a-5p, which in turn activates c-MYC activity promoting breast cancer. CircPVT1 is highly enriched in exosomal vesicles, and acts as an immunomodulator by shuttling between malignant cells (27). However, the role of circPVT1 in tumors remains unknown, particularly in ccRCC immunity. In our study, we found that circPVT1 was highly expressed in ccRCC. *In vitro* functional assays and *in vivo* xenograft experiments revealed the pivotal role of circPVT1 in ccRCC development and metastasis.

During tumor progression, M1-type macrophages gradually transform into M2-type macrophages (28, 29). These M2 macrophages create an immunosuppressive environment that promotes tumor growth through mechanisms such as tumor treatment resistance and distant metastasis (30). Thus, we sought to understand the effect of circPVT1 on macrophage polarization. WB assays showed that circPVT1 knockdown promotes macrophage conversion to the M1 subtype. ELISA experiments showed that circPVT1 knockdown inhibits the secretion of IL-4, IL-10, and TGF-β from tumor cells. The above results indicate that inhibiting circPVT1 in tumor cells may promote the M1 polarization of macrophages through the secretion of cytokines. The critical role of circPVT1 in the progression of ccRCC was confirmed through our aforementioned analyses. Therefore, the development of drugs that inhibit circPVT1 expression was deemed crucial.

The utilization of nanoparticles (NPs) as gene delivery vectors represents a future direction in gene therapy for tumor diseases (31, 32). In recent years, breakthroughs in nanomedicine have revolutionized the landscape of nanocarrier-based drug delivery. Especially, liposomes and PDA NPs, with their excellent biocompatibility and biodegradability, are considered promising candidates (33, 34). The transmembrane glycoprotein MUC12, which overexpressed in colorectal carcinoma, hepatocellular carcinoma, and RCC, has been validated as a promising surface biomarker for RCC targeting (35, 36).

Considering these findings, we proposed to rationally design liposomal PDA NPs to target MUC12 on the surface of RCC, and thus, deliver the si-circPVT1 encapsulated in the NPs to exert their intracellular functions. We developed a nanostructure with liposomes and PDA as carriers to deliver si-circPVT1, which in turn could be used for therapeutic purposes in RCC. Based on the aforementioned research findings, we proposed the rational design of liposomal PDA NPs to target MUC12 on the surface of RCC and deliver the si-circPVT1 to exert their intracellular functions. Although no significant weight loss or damage to major organs was observed in mice during the treatment period, which suggests a certain level of tolerability. This parameter alone is insufficient for a comprehensive assessment of the *in vivo* safety of SCLPM-NPs Additional indicators, such as routine blood tests and serum biochemical parameters, were not evaluated in the current study. Therefore, the biosafety profile of the nanotherapeutic system remains to be fully characterized. Future investigations should incorporate a more extensive panel of safety assessments to validate the clinical translational potential of SCLPM-NPs.

In this study, circPVT acted as a tumor promoter circRNA which facilitated ccRCC progression and metastasis through the EMT signaling pathway during the progression of ccRCC. Subsequently, single-cell sequencing results indicated increased macrophage infiltration in the circPVT1-high expression group. ELISA and flow cytometry results confirmed that the circPVT1 knockdown group induces macrophage polarization toward the M1 phenotype by suppressing the secretion of IL-4, IL-10, and TGF-β. To summarize, circPVT1 may be a predictor of ccRCC immune evasion and a potential therapeutic target.

## Data Availability

The original contributions presented in the study are included in the article/**Supplementary Material**. Further inquiries can be directed to the corresponding authors.

## References

[1] Zhang M Liu H Yin R Xu J Fan S Qian X . PRMT5-mediated arginine methylation of ACSL4 attenuates its stability and suppresses ferroptosis in renal cancer. Res (Washington DC). (2025) 8:789. doi: 10.34133/research.0789. PMID: 40756764 PMC12314280

[2] Rini BI Campbell SC Escudier B . Renal cell carcinoma. Lancet. (2009) 373:1119–32. doi: 10.1002/9781118592168.ch17. PMID: 19269025

[3] Mao W Wang K Zhang W Chen S Xie J Zheng Z . Transfection with plasmid-encoding lncRNA-SLERCC nanoparticle-mediated delivery suppressed tumor progression in renal cell carcinoma. J Exp Clin Cancer Research: CR. (2022) 41:252. doi: 10.1186/s13046-022-02467-2. PMID: 35986402 PMC9389749

[4] Hsieh JJ Purdue MP Signoretti S Swanton C Albiges L Schmidinger M . Renal cell carcinoma. Nat Rev Dis Primers. (2017) 3:17009. doi: 10.1038/nrdp.2017.9. PMID: 28276433 PMC5936048

[5] Choueiri TK Motzer RJ . Systemic therapy for metastatic renal-cell carcinoma. N Engl J Med. (2017) 376:354–66. doi: 10.1056/nejmra1601333. PMID: 28121507

[6] Pan XW Zheng HF Li MC Dong KQ Tang YF Chen JX . Unveiling the mechanisms of CEBPD-orchestrated TAM polarization through RGS2/PAR2 pathway and its impact on ICB efficacy in clear cell renal cell carcinoma. J Immunother Cancer. (2025) 13(7):e010898. doi: 10.1136/jitc-2024-010898. PMID: 40659446 PMC12258302

[7] Potter SS . Single-cell RNA sequencing for the study of development, physiology and disease. Nat Rev Nephrol. (2018) 14:479–92. doi: 10.1038/s41581-018-0021-7. PMID: 29789704 PMC6070143

[8] Saliba AE Westermann AJ Gorski SA Vogel J . Single-cell RNA-seq: advances and future challenges. Nucleic Acids Res. (2014) 42:8845–60. doi: 10.1093/nar/gku555. PMID: 25053837 PMC4132710

[9] Lei T Chen R Zhang S Chen Y . Self-supervised deep clustering of single-cell RNA-seq data to hierarchically detect rare cell populations. Brief Bioinform. (2023) 24(6):bbad335. doi: 10.1093/bib/bbad335. PMID: 37769630 PMC10539043

[10] Bagnoli JW Ziegenhain C Janjic A Wange LE Vieth B Parekh S . Sensitive and powerful single-cell RNA sequencing using mcSCRB-seq. Nat Commun. (2018) 9:2937. doi: 10.1038/s41467-018-05347-6. PMID: 30050112 PMC6062574

[11] Moses L Pachter L . Museum of spatial transcriptomics. Nat Methods. (2022) 19:534–46. doi: 10.1038/s41592-022-01409-2. PMID: 35273392

[12] Longo SK Guo MG Ji AL Khavari PA . Integrating single-cell and spatial transcriptomics to elucidate intercellular tissue dynamics. Nat Rev Genet. (2021) 22:627–44. doi: 10.1038/s41576-021-00370-8. PMID: 34145435 PMC9888017

[13] Song X Zhu Y Geng W Jiao J Liu H Chen R . Spatial and single-cell transcriptomics reveal cellular heterogeneity and a novel cancer-promoting Treg cell subset in human clear-cell renal cell carcinoma. J Immunother Cancer. (2025) 13(1):e010183. doi: 10.1136/jitc-2024-010183. PMID: 39755578 PMC11748785

[14] Zhu S Chen Y Lin H Lu J Pan Y Li G . SenExo-cCCT2 reprograms senescence response and anti-tumor immunity following FOLFIRINOX chemotherapy in pancreatic ductal adenocarcinoma. Adv Sci (Weinh). (2025) 12(38):e08431. doi: 10.1002/advs.202508431. PMID: 40686389 PMC12520514

[15] Northoff BH Herbst A Wenk C Weindl L Gabel G Brezski A . Circular RNAs increase during vascular cell differentiation and are biomarkers for vascular disease. Cardiovasc Res. (2025) 121:405–23. doi: 10.1093/cvr/cvaf013. PMID: 39901821 PMC12038242

[16] Yu X Ding P Guo M Tang X Wang Z Zhang Y . Extracellular vesicle-mediated delivery of circp53 suppresses the progression of multiple cancers by activating the CypD/TRAP/HSP90 pathway. Exp Mol Med. (2025) 57(8):1711–26. doi: 10.1038/s12276-025-01506-0. PMID: 40744997 PMC12411616

[17] Dong Y Gao Q Chen Y Zhang Z Du Y Liu Y . Identification of CircRNA signature associated with tumor immune infiltration to predict therapeutic efficacy of immunotherapy. Nat Commun. (2023) 14:2540. doi: 10.1038/s41467-023-38232-y. PMID: 37137884 PMC10156742

[18] Zhang H Tao T Ji J Zhao T Sun S Zhang L . CircPVT1 promotes lung metastasis and tumor progression in renal cell carcinoma by encoding the cP104aa peptide and targeting EIF4A3. Adv Sci (Weinh). (2025) 12:e01211. doi: 10.1002/advs.202501211. PMID: 40966379 PMC12752572

[19] Mao W Xu K Wang K Zhang H Ji J Geng J . Single-cell RNA sequencing and spatial transcriptomics of bladder Ewing sarcoma. iScience. (2024) 27:110921. doi: 10.1016/j.isci.2024.110921. PMID: 39386756 PMC11462044

[20] Pastushenko I Brisebarre A Sifrim A Fioramonti M Revenco T Boumahdi S . Identification of the tumour transition states occurring during EMT. Nature. (2018) 556:463–8. doi: 10.1038/s41586-018-0040-3. PMID: 29670281

[21] Cheng K Cai N Zhu J Yang X Liang H Zhang W . Tumor-associated macrophages in liver cancer: From mechanisms to therapy. Cancer Commun (Lond). (2022) 42:1112–40. doi: 10.1002/cac2.12345. PMID: 36069342 PMC9648394

[22] Guo B Cen H Tan X Ke Q . Meta-analysis of the prognostic and clinical value of tumor-associated macrophages in adult classical Hodgkin lymphoma. BMC Med. (2016) 14:159. doi: 10.1186/s12916-016-0711-6. PMID: 27745550 PMC5066288

[23] Shen H Liu J Chen S Ma X Ying Y Li J . Prognostic value of tumor-associated macrophages in clear cell renal cell carcinoma: A systematic review and meta-analysis. Front Oncol. (2021) 11:657318. doi: 10.3389/fonc.2021.657318. PMID: 34026635 PMC8136289

[24] Lawrence T Natoli G . Transcriptional regulation of macrophage polarization: enabling diversity with identity. Nat Rev Immunol. (2011) 11:750–61. doi: 10.1038/nri3088. PMID: 22025054

[25] Yang J Wu S He M . Extracellular vesicle-mediated delivery of mitochondrial circRNA MTCO2 protects against cerebral ischemia by modulating mPTP-dependent ferroptosis. Redox Biol. (2025) 86:103806. doi: 10.34133/research.1232. PMID: 40768899 PMC12344986

[26] Wang Y Lin L Wang X Li J Pan Q Kou H . Synergically enhanced anti-tumor immunity of *in vivo* panCAR by circRNA vaccine boosting. Cell Rep Med. (2025) 6:102250. doi: 10.1016/j.xcrm.2025.102250. PMID: 40712575 PMC12432353

[27] Ghetti M Vannini I Bochicchio MT Azzali I Ledda L Marconi G . Uncovering the expression of circPVT1 in the extracellular vesicles of acute myeloid leukemia patients. BioMed Pharmacother. (2023) 165:115235. doi: 10.1016/j.biopha.2023.115235. PMID: 37536029

[28] Belgiovine C D'Incalci M Allavena P Frapolli R . Tumor-associated macrophages and anti-tumor therapies: complex links. Cell Mol Life Sci. (2016) 73:2411–24. doi: 10.1007/s00018-016-2166-5. PMID: 26956893 PMC11108407

[29] Li M Lu L Xiao Q Maalim AA Nie B Liu Y . Bioengineer mesenchymal stem cell for treatment of glioma by IL-12 mediated microenvironment reprogramming and nCD47-SLAMF7 mediated phagocytosis regulation of macrophages. Explor (Beijing). (2024) 4:20240027. doi: 10.22541/au.170994437.77141754/v1. PMID: 39713206 PMC11657999

[30] Zhang M Liu ZZ Aoshima K Cai WL Sun H Xu T . CECR2 drives breast cancer metastasis by promoting NF-kappaB signaling and macrophage-mediated immune suppression. Sci Transl Med. (2022) 14:eabf5473. doi: 10.1126/scitranslmed.abf5473. PMID: 35108062 PMC9003667

[31] Forbes DC Peppas NA . Oral delivery of small RNA and DNA. J Control Release. (2012) 162:438–45. doi: 10.1016/j.jconrel.2012.06.037. PMID: 22771979

[32] Qiao E Qiu F Guo X Ding J Chen X Zheng L . Biomaterial-mediated modulation of tumor microenvironment and therapeutic sensitization. EngMedicine. (2025) 2:100092. doi: 10.1016/j.engmed.2025.100092. PMID: 41916819

[33] Seth A Gholami Derami H Gupta P Wang Z Rathi P Gupta R . Polydopamine-mesoporous silica core-shell nanoparticles for combined photothermal immunotherapy. ACS Appl Mater Interfaces. (2020) 12:42499–510. doi: 10.1021/acsami.0c10781. PMID: 32838525 PMC7942218

[34] Gholami Derami H Gupta P Weng KC Seth A Gupta R Silva JR . Reversible photothermal modulation of electrical activity of excitable cells using polydopamine nanoparticles. Adv Mater. (2021) 33:e2008809. doi: 10.1002/adma.202008809. PMID: 34216406 PMC8363531

[35] Zhang B Chu W Wen F Zhang L Sun L Hu B . Dysregulation of long non-coding RNAs and mRNAs in plasma of clear cell renal cell carcinoma patients using microarray and bioinformatic analysis. Front Oncol. (2020) 10:559730. doi: 10.3389/fonc.2020.559730. PMID: 33330027 PMC7729199

[36] Huang H Hu Y Guo L Wen Z . Integrated bioinformatics analyses of key genes involved in hepatocellular carcinoma immunosuppression. Oncol Lett. (2021) 22:830. doi: 10.3892/ol.2021.13091. PMID: 34691257 PMC8527569

