# Supplementary material for: circPVT1 regulates EMT and induces macrophage polarization to promotes the progression of renal cell carcinoma
Source: Front Immunol. 2026 Apr 21;17:1760058. doi: 10.3389/fimmu.2026.1760058 (PMC13139081; doi:10.3389/fimmu.2026.1760058)
Supplement: Supplementary file 1 [file DataSheet1.docx]

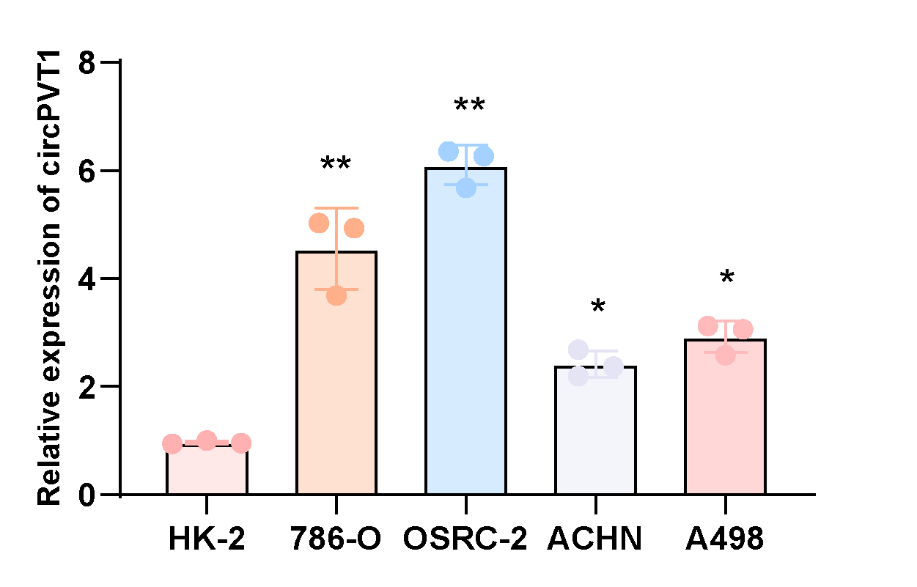


**Figure S1.** Expression levels of circPVT1 in RCC cell lines quantified through qRT-PCR.


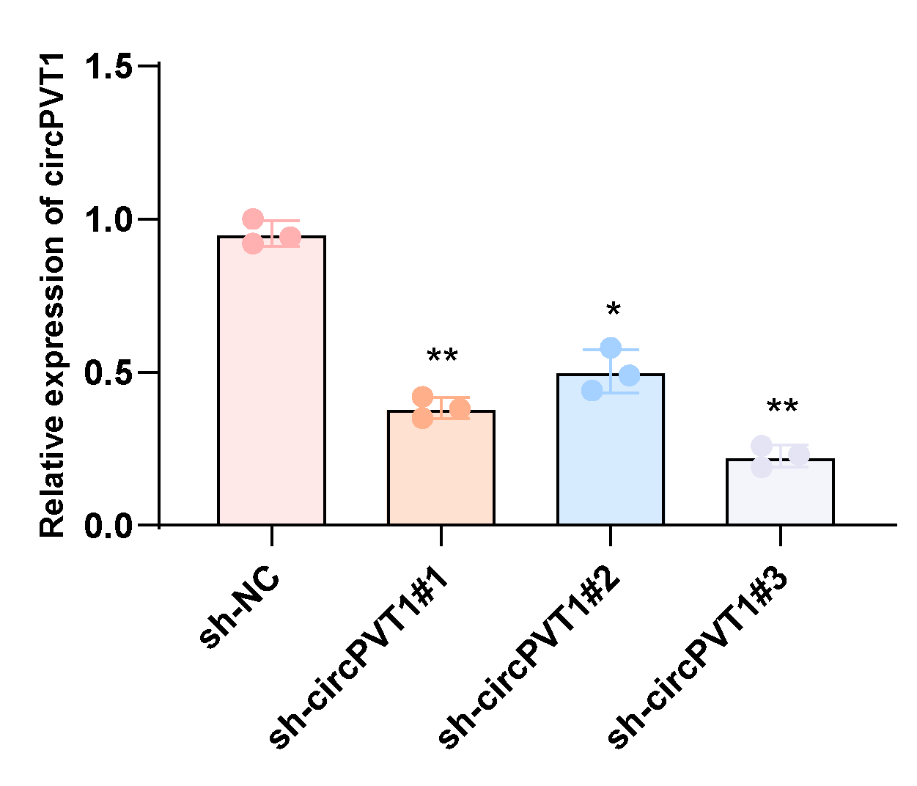


**Figure S2.** Transfection of sh-NC, sh-circPVT1#1, sh-circPVT1#2 and sh-circPVT1#3. N.S.: not significant. Statistical significance is indicated (*P<0.05 and **P<0.01) by Student's t-test or ANOVA.


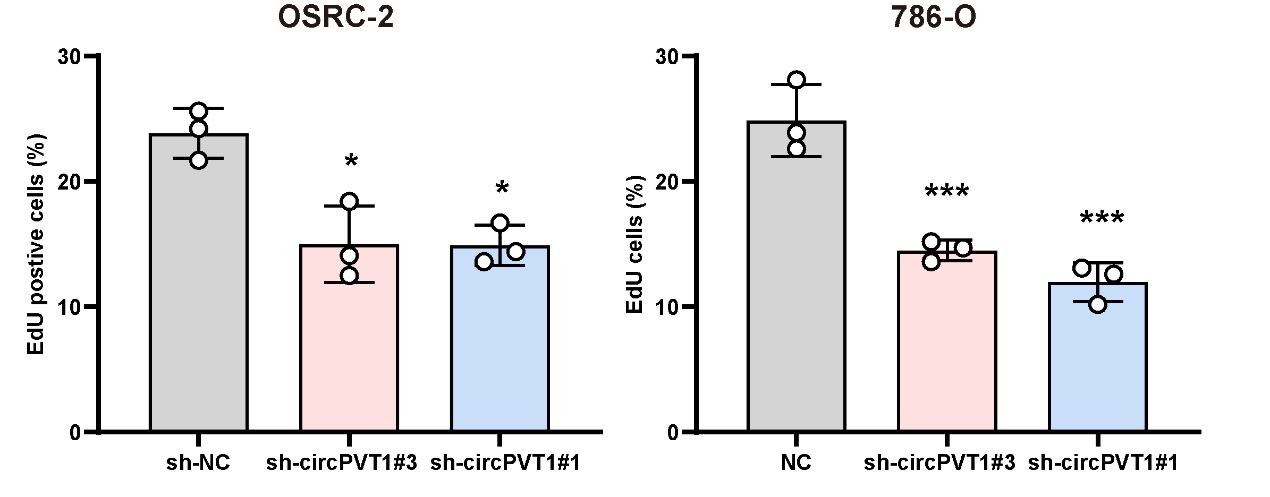


**Figure S3.** The corresponding quantitative analysis of EdU positive cells.


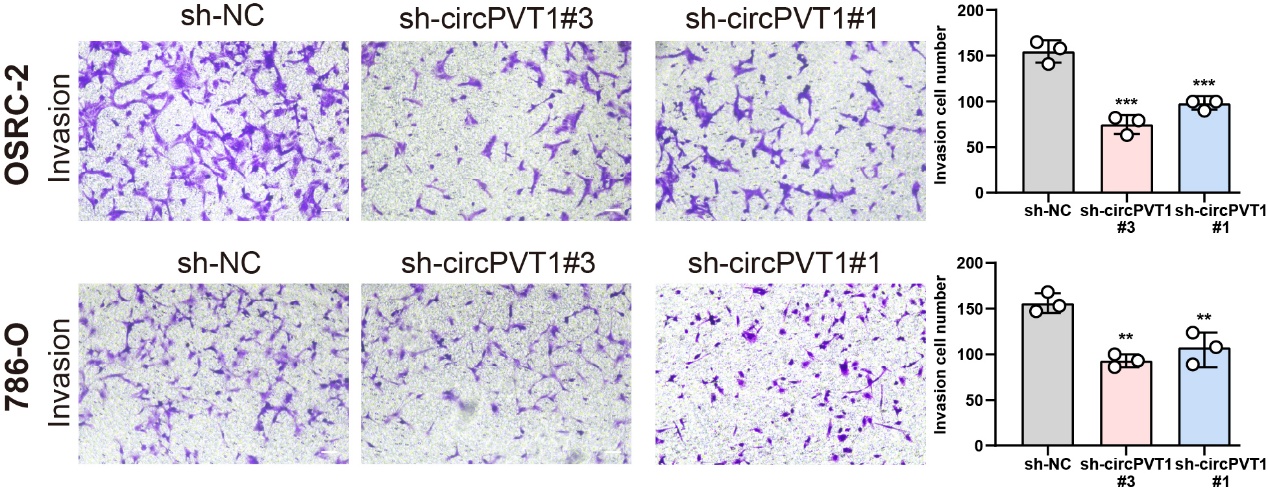


**Figure S4.** Transwell invasion assay was performed to detect the effects of circPVT1 on the invasion of OSRC-2 and 786-O cells. Scale bar: 100 μm. Statistical significance is indicated (**P<0.01, ***P<0.001) by Student's t-test or ANOVA.


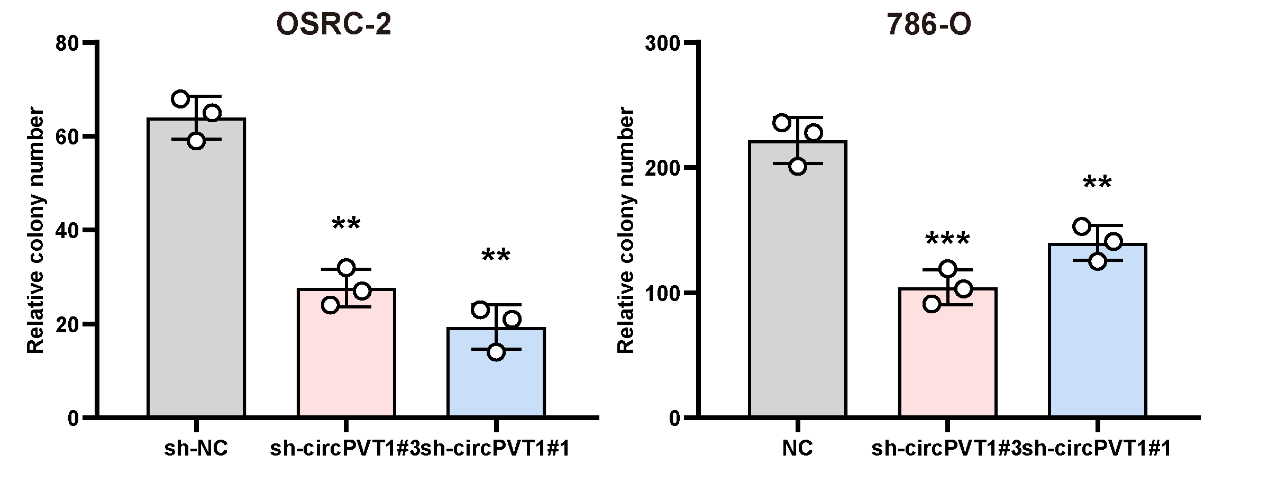


**Figure S5.** The corresponding quantitative analysis of cloning number.


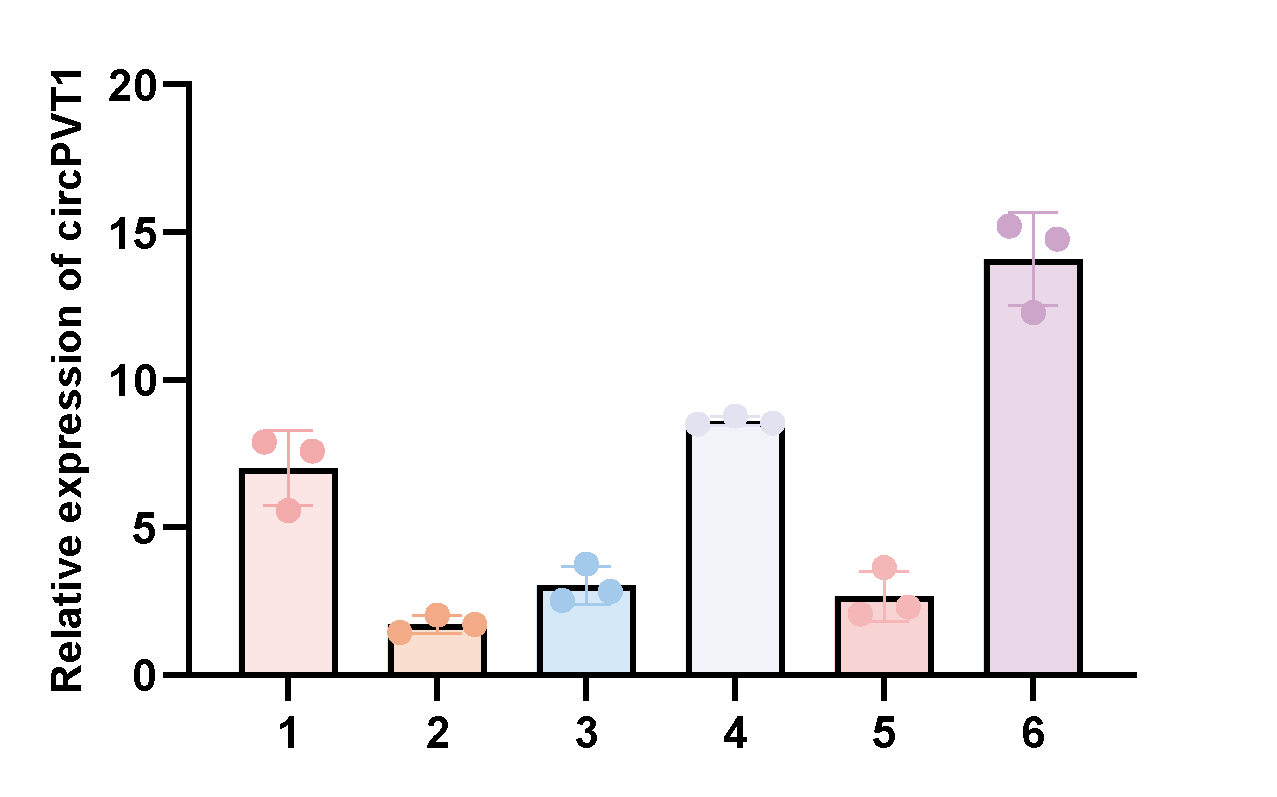


**Figure S6.** Expression levels of circPVT1 in six samples quantified through qRT-PCR.


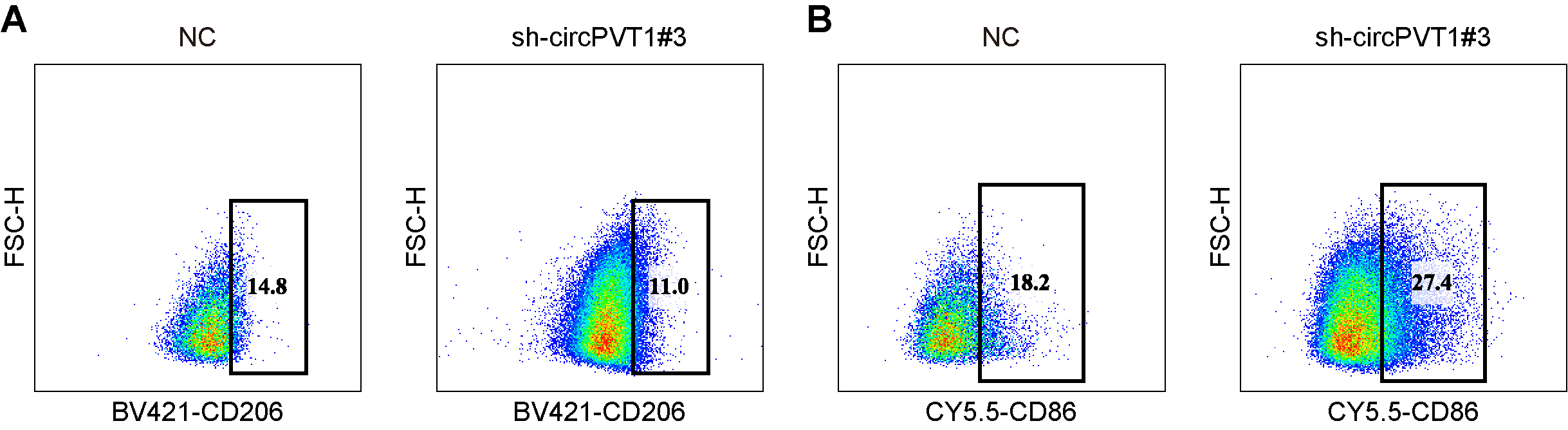


**Figure S7. A, B)** Representative flow cytometry plots show the percentage of M2 and M1 macrophage.


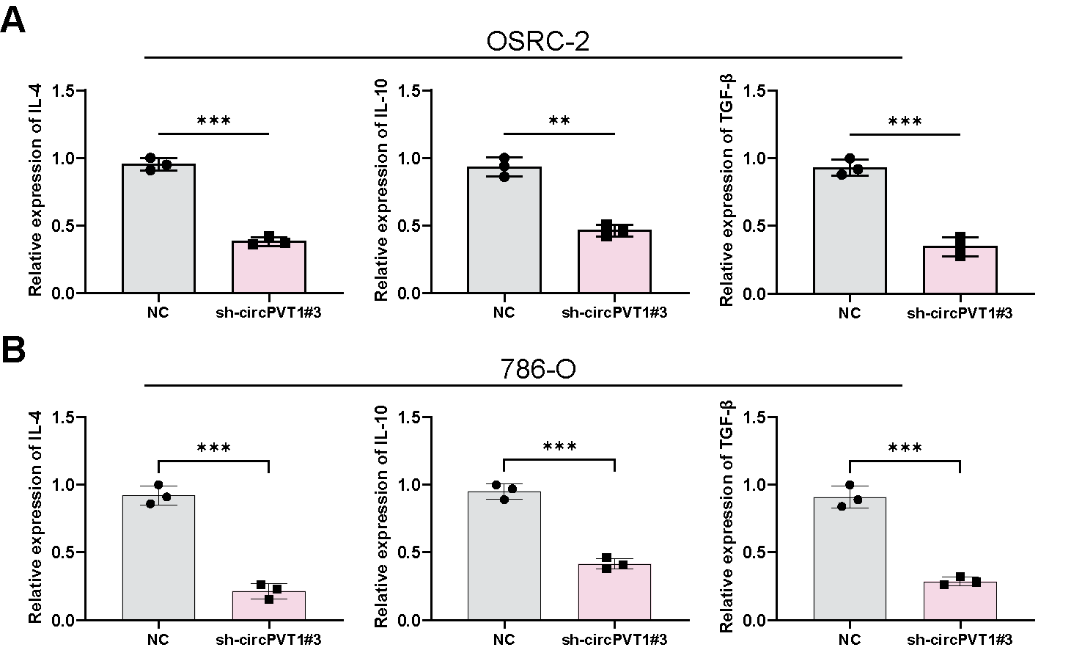


**Figure S8.** ELISA assay was performed to detect IL-4, IL-10 and TGF-β secreted by OSRC-2 and 786-O cells. A) OSRC-2 cells. B) 786-O cells. Statistical significance is indicated (**P<0.01, ***P<0.001) by Student's t-test or ANOVA.


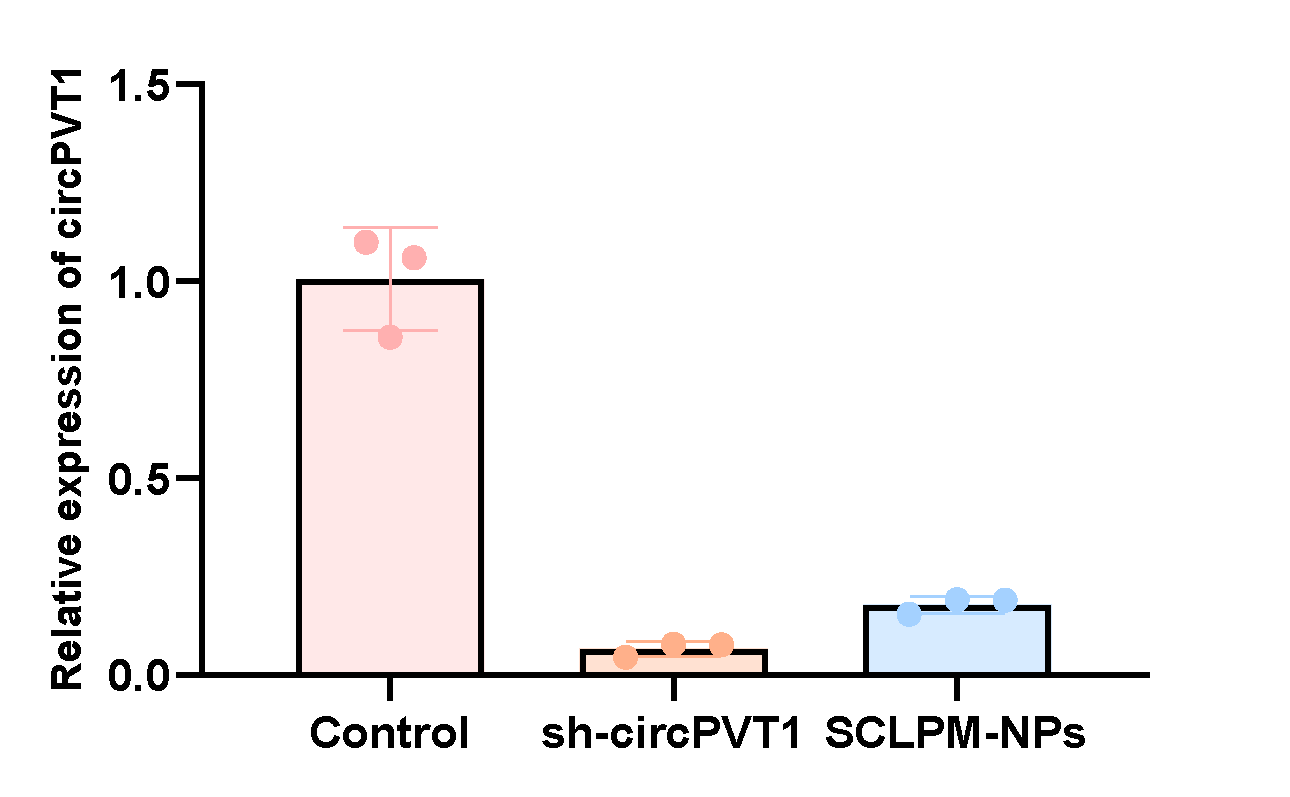


**Figure S9.** Expression levels of circPVT1 in the control, sh-circPVT1 and SCLM NPs groups.
